# Fitness benefits and costs of shelters to the sea urchin *Glyptocidaris crenularis*

**DOI:** 10.7717/peerj.8886

**Published:** 2020-04-20

**Authors:** Xiaomei Chi, Jiangnan Sun, Yushi Yu, Jia Luo, Bao Zhao, Feng Han, Yaqing Chang, Chong Zhao

**Affiliations:** Key Laboratory of Mariculture & Stock Enhancement in North China’s Sea, Ministry of Agriculture and Rural Affairs, Dalian Ocean University, Dalian, China

**Keywords:** Sea urchin, Aristotle’s lantern reflex, Shelter, Behavior, Growth

## Abstract

Understanding the ecological role of shelters is greatly hampered by the scarcity of long-term laboratory experiments on the trade-off between fitness benefits and costs. This lack probably leads to an underestimation of the negative and/or positive effects on behaviors and growth of marine invertebrates in benthic ecosystems. Although our previous study revealed a significant effect on fitness-related traits of *Glyptocidaris crenularis* after 31 months, the present study extended it and investigated fitness benefits and/or costs of long-term sheltering on sea urchins to over 7 years. The present long-term study suggests that the previously reported reduction in feeding rate probably resulted from a reduction in reflexive feeding motions (Aristotle’s lantern reflex) rather than changes in foraging behavior. Actively seeking sheltering behavior was negatively impacted in individuals with continuous access to shelters. However, covering and righting behaviors did not differ in sheltered sea urchins, indicating that these behaviors are maintained to escape from adverse environments regardless of shelter. Body size of sea urchins in the group with shelters was significantly lower than those without shelters after 7 years. Weights of gonads and gut were not significantly different after 7 years despite previous observations of differences after ~2.5 years. The present study provides valuable information on the trade-off between fitness benefits and costs to sea urchins residing in shelters. However, the present study is only a laboratory investigation for one urchin species (*G. crenularis*) which does not consider the complexity of natural environments. Field studies should be carried out with *G. crenularis* and other sea urchin species, before a more universal conclusion can be drawn.

## Introduction

Besides evolutionary adaptation, acclimation is important for fitness benefits of marine invertebrates (Sanford & Kelly, 2011; Foo & Byrne, 2016). Alternatively, negative consequences probably exist as fitness costs. The trade-off between fitness benefits and costs probably shapes the evolutionary process of marine invertebrates in different environments (Abarca & Boege, 2011).

Shelters are a representative habitat condition in marine benthic ecosystems. Marine invertebrates escape from predation and water turbulence by inhabiting crevices, cracks, burrows and spaces under rocks and boulders (Agatsuma, 2013; Tamaki, Muraoka & Inoue, 2018). Consequently, shelters are important for the fitness benefits of marine invertebrates in benthic ecosystems. However, shelters probably have fitness costs. For example, *Strongylocentrotus intermedius* preferred covering behavior to sheltering behavior when covering material was outside the shelter, indicating covering and sheltering behaviors probably have different consequences on fitness traits of sea urchins (Zhao et al., 2014). Furthermore, a trade-off exists between foraging and sheltering behaviors of *S. intermedius* (Zhao et al., 2013). We hypothesized the foraging behavior of *Glyptocidaris crenularis* would be affected in the long-term shelter conditions. Understanding the ecological role of shelters is greatly hampered by the scarcity of long-term laboratory experiments on the trade-off between fitness benefits and costs. This lack probably leads to an underestimation of the negative and/or positive effects on behaviors and growth of marine invertebrates in benthic ecosystems.

The sea urchin *G. crenularis* is an ecologically important marine invertebrate in structuring marine benthic ecosystems (Chang et al., 2004). We previously found that sheltered *G. crenularis* in the laboratory showed lower test diameter, wet body weight, test weight, lantern weight, gut weight and gonad weights in comparison to those in non-sheltering conditions, during a 2.5 years census (Zhao, Bao & Chang, 2016). Like other echinoids, *G. crenularis* display a number of fitness related behaviors, including Aristotle’s lantern reflex, foraging, righting, covering and sheltering behaviors. Aristotle’s lantern reflex demonstrates a sea urchin’s ability to grasp food with their teeth (Brothers & McClintock, 2015). In addition, foraging is a behavior of sea urchins to detect and locate chemicals released from potential food sources and move toward them within short distances (Ceccherelli et al., 2009). Righting behavior refers to the resumption of an inverted individual to the posture with the aboral side up (Hyman, 1955). It is essential for sea urchins to escape from predators and the effects of turbulence (Brothers & McClintock, 2015). Covering is the behavior of a sea urchin in which they hold shells, stones, onto their dorsal surface (Verling, Crook & Barnes, 2002; Pawson & Pawson, 2013). Sheltering behavior is a behavioral habit of sea urchins in which they inhabit refuge habitats, for example, cracks, pockets and crevices, for the avoidance against predators. Thus, *G. crenularis* is an ideal research model to investigate the effects on behaviors and growth of sea urchins in the shelter conditions over long duration in benthic ecosystems. The main purposes of the present study are to investigate (1) whether Aristotle’s lantern reflex and/or foraging behavior can account for the potential fitness costs of feeding, (2) whether sheltering behavior is negatively impacted in the shelter conditions over long durations (7 years), (3) whether covering and righting behaviors decrease for sea urchins of the group with shelters, (4) whether body size and organs (gonads and guts) of *G. crenularis* are affected in shelter conditions over 7 years.

## Methods

### Sea urchins and experimental design

This present study is an extension of our previous report (Zhao, Bao & Chang, 2016). The experiment lasted for over 7 years from March 2011 to June 2018. A summary of the experimental design is provided below. Behavioral experiments associated with the shelter conditions were performed as follows: in the group with shelters, bricks (measuring 23 × 17 × 11 cm and 23 × 11 × 5 cm) were arranged in four tanks (measuring 85 × 52 × 60 cm) to make one large (17 × 12 cm), 18 small (4 × 4 cm) and several irregular openings into the shelter (Zhao, Bao & Chang, 2016). In the group without shelters, there were no bricks in another four tanks with the same dimensions of the sheltered treatment tanks (Zhao, Bao & Chang, 2016). Sea urchins were ad libitum fed macroalgae *Saccharina japonica*, *Undaria pinafida* and *Ulva pertusa* according to availability. Seawater was changed every 3 days.

At the end of the experiment, five sea urchins were randomly chosen for the following measurements of fitness related traits as follows (*N* = 5). Righting, Aristotle’s lantern reflex, foraging and covering behavioral experiment were measured under similar illumination and water quality conditions in order to avoid potential non-experimental influences. We changed the seawater and washed the experimental holding tanks for each behavioral experiment. To avoid potential fatigue effects, behavioral experiments were carried out every 3 days after the long duration of shelter conditions (7 years).

### Righting behavior

**Figure 1:**
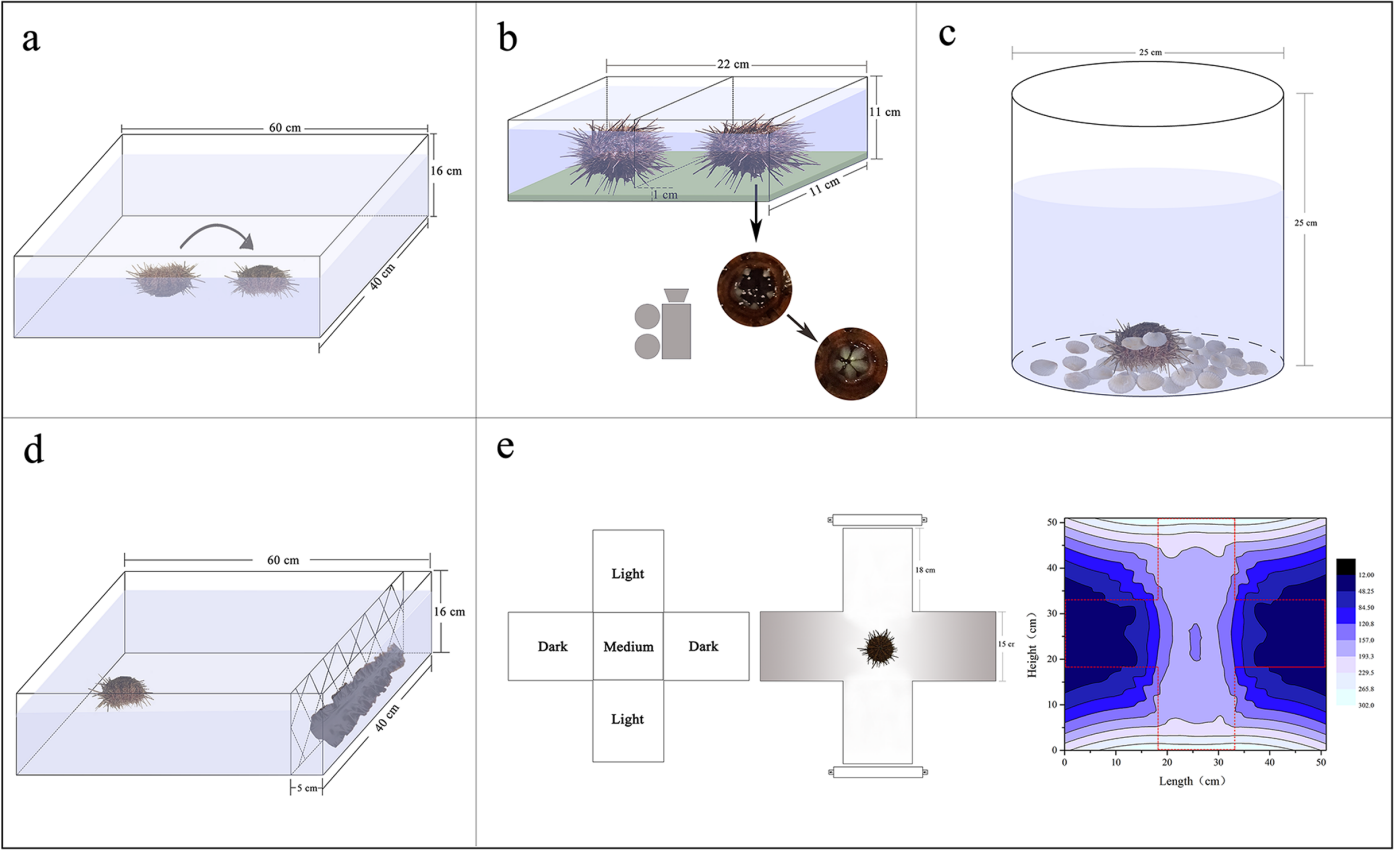
Diagrams showing the experimental devices for the measurements of righting behavior (A), Aristotle’s lantern reflex (B), covering behavior (C), foraging behavior (D) and sheltering behavior (E).

Sea urchins were individually distributed on the bottom of an experimental tank (measuring 60 × 40 × 16 cm) with their oral side up. Righting response time refers to time required for inverted *G. crenularis* to turn themselves over with the oral side down (*N* = 5, Fig. 1A). If the sea urchin failed to right itself within 10 min, we stopped the experiment and recorded 600 s as the righting response time (Zhang et al., 2017).

### Aristotle’s lantern reflex

Aristotle’s lantern reflex response refers to one cycle from opening to closing of the teeth (Brothers & McClintock, 2015). A simple experimental tank was designed for the measurement of Aristotle’s lantern reflex. There were two small compartments (each measuring 11 × 11 × 11 cm) with a kelp-based film on the bottom (Fig. 1B). The configuration method of kelp-based film is as follows (Ding et al., 2020): We weighed 2 g kelp powder and 3 g agar powder using an electric balance (G & G Co., Arlington, TX, USA). The kelp powder was added to 50 mL filtered seawater in a beaker, mixed and then filtered using a fine silk net (mesh size: 25 μm). We subsequently added the agar powder, mixed and heated it in a microwave for 60 s. The mixed liquid was poured into the tanks and cooled. Four sea urchins from each group with or without shelters were carefully placed into the devices, respectively (*N* = 4, Fig. 1B). The kelp-based film was changed for each trial. The number of lantern reflex cycles was recorded over 5 min using a digital camera (Legria HF20; Canon, Tokyo, Japan) positioned under the holding device (Fig. 1B).

### Covering behavior

Covering behavior of an individual in a plastic bucket (25 cm of diameter, 25 cm of height) was measured (*N* = 5, Fig. 1C). Forty-eight shells of small scallops *Patinopecten yessoensis* (20 ± 1 mm of shell height) were evenly distributed around the bottom of each plastic bucket as covering material (Fig. 1C). The shells were washed for each experiment to avoid potential non-experimental effects. Time to first covering and number of shells used for covering were recorded over 1 h. We took pictures for each experimental sea urchin using a digital camera (Legria HF20; Canon, Tokyo, Japan), measured and calculated the percentage of covered areas using software ImageJ.

### Foraging behavior

A simple experimental tank was designed for the measurement of foraging behavior of sea urchins (measuring 60 × 40 × 16 cm). Two hundred grams of brown alage (*S. japonica*) was placed on one side of the tank and a single sea urchin on the opposite side (Fig. 1D). We washed the *S. japonica* for each experiment to avoid potential influences. Foraging time refers to the time required for a sea urchin to reach the algae. Total distance of movement, velocity, acceleration were also recorded using a digital recorder (Legria HF20; Canon, Tokyo, Japan), and subsequently calculated using software ImageJ.

### Sheltering behavior

An aquatic dark/light plus-shaped maze was designed for the measurement of sheltering behavior, comprising two light arms, two sheltered arms and a starting (“medium”) zone with seawater of 80 mm depth (Fig. 1E; Fossat, 2014). Light intensities under water were 160.3–296.7 lx in the lighted arms, 13.6–47.7 lx in the sheltered arms and 153.3–159.2 lx in the medium zone measured by an illumination photometer (Digital Luxmeter, China) (Fig. 1E). Sea urchins were individually placed in the medium of the tank. Movement of sea urchins was recorded for 30 min using a digital video recorder (Legria HF20; Canon, Tokyo, Japan). The length of time of sea urchins spent in the sheltered areas was calculated using software ImageJ. Sheltering behavior of individuals was measured under the light intensity for each group.

### Body size and organ weights

Body size and organ weight were recorded at the end of the experiment (Zhao et al., 2018b), which are briefly described as follows:

Test diameter and test height were measured by a digital vernier calipers (Mahr Co., Göttingen, Germany). Test height:test diameter ratio was subsequently calculated. Individuals were weighed using an electric balance (G & G Co., Arlington, TX, USA). Sea urchins were dissected after the measurement of body size. The organs were the test with spines, Aristotle’s lantern, gut and gonads. The gut was dissected out using tweezers and the contents removed. The gonads were removed out using a spoon (Jones et al., 2010; Ding et al., 2020). Lantern length and test thickness were measured using a digital vernier caliper according to the method of Byrne et al. (2014). Wet weight of test, lantern, gonads and gut were weighed to the nearest 0.01 g using an electric balance (G & G Co., Arlington, TX, USA).

### Statistical analysis

All data were tested for normal distribution and homogeneity of variance using Kolmogorov–Smirnov test and Levene test, respectively. Independent sampled *t*-tests were performed to detect differences in behaviors and growth between the two experimental groups. All data analyses were conducted with SPSS 21 statistical software. A probability level of *P* < 0.05 was considered as significant.

## Results

### Righting behavior

**Figure 2:**
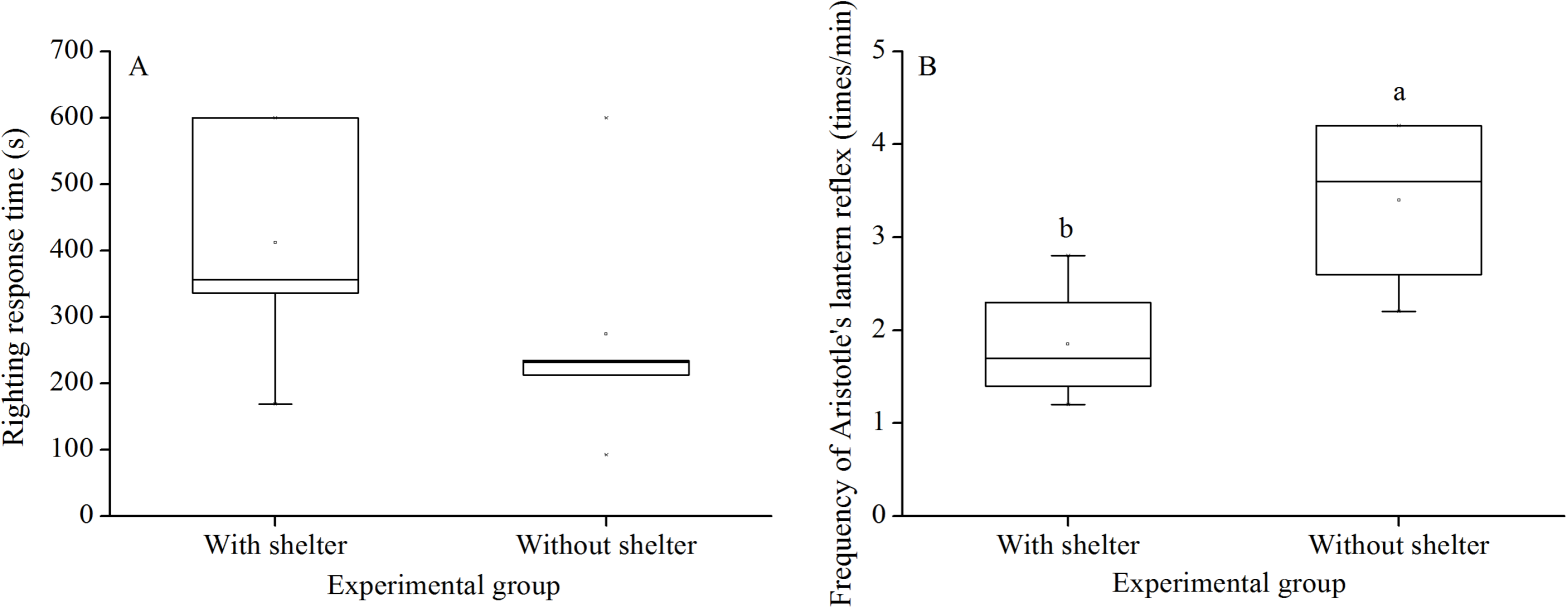
Righting response time (A) and Aristotle’s lantern reflex (B) of *Glyptocidaris crenularis* in different experimental groups 5 min after the beginning of observations (*N* = 5 for righting response, *N* = 4 for Aristotle’s lantern reaction mean ± SD). Letters above the bars represent significance (*P* < 0.05).

Righting response time showed no significant difference between *G. crenularis* in the groups with and without shelters (*P* = 0.281, Fig. 2A).

### Aristotle’s lantern reflex

Aristotle´s lantern reflex of groups without shelters (3.40 ± 0.98 times/min) was significantly higher than that of groups with shelters (1.85 ± 0.68 times/min) (*P* = 0.041, Fig. 2B).

### Covering behavior

**Figure 3:**
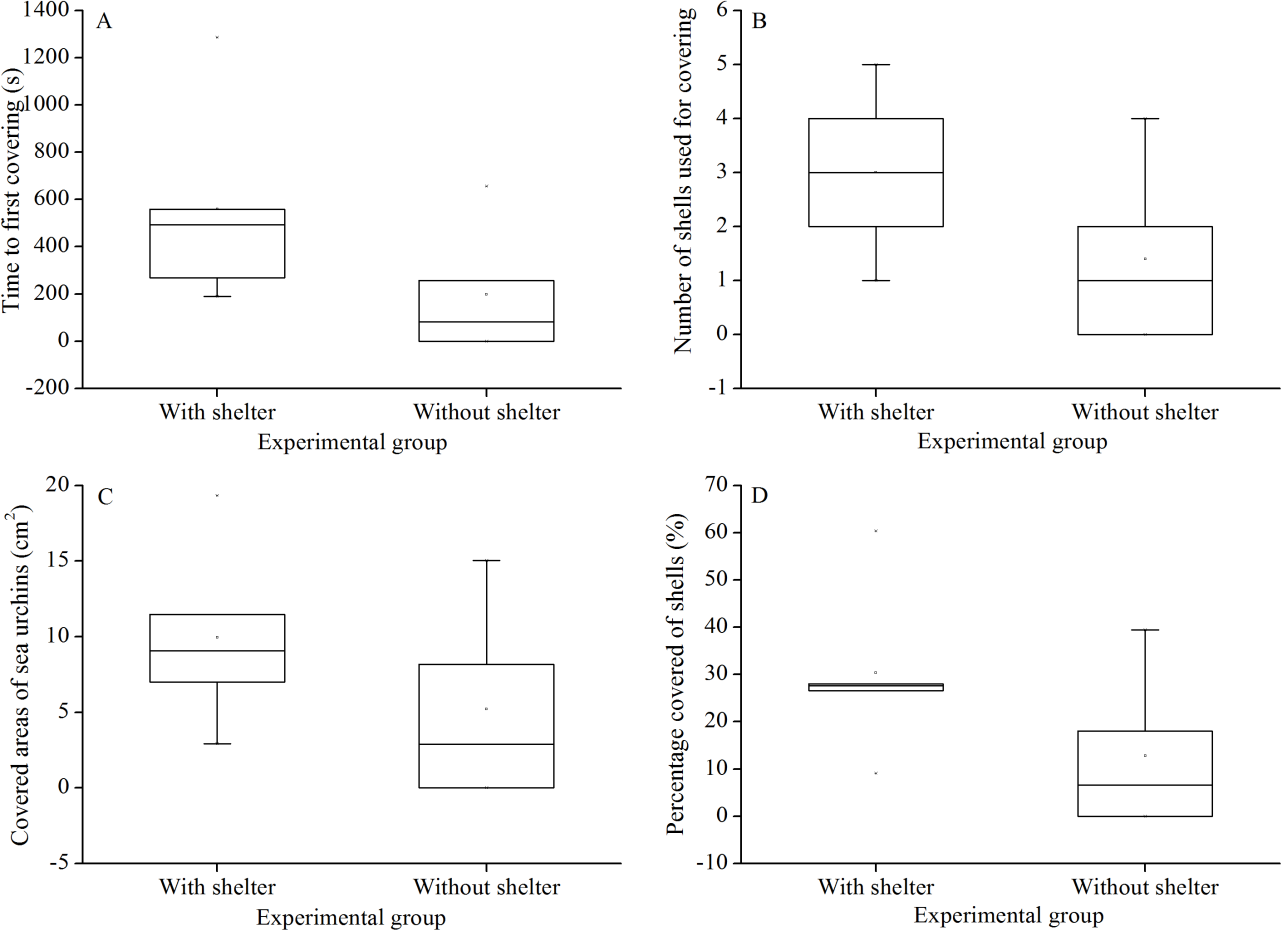
Time to first covering (A), number of shells used for covering (B), covered areas of sea urchins (C) and percentage of covered areas (D) of Glyptocidaris crenularis in different experimental groups in 1 hour after the beginning of experiment (*N* = 5, mean ± SD).

There were no significant differences in time to first covering (*P* = 0.156, Fig. 3A), number of shells used for covering (*P* = 0.158, Fig. 3B), covered areas of sea urchins (*P* = 0.267, Fig. 3C) and percentage of covered areas (*P* = 0.154, Fig. 3D) between the two treatment groups.

### Foraging behavior

**Figure 4:**
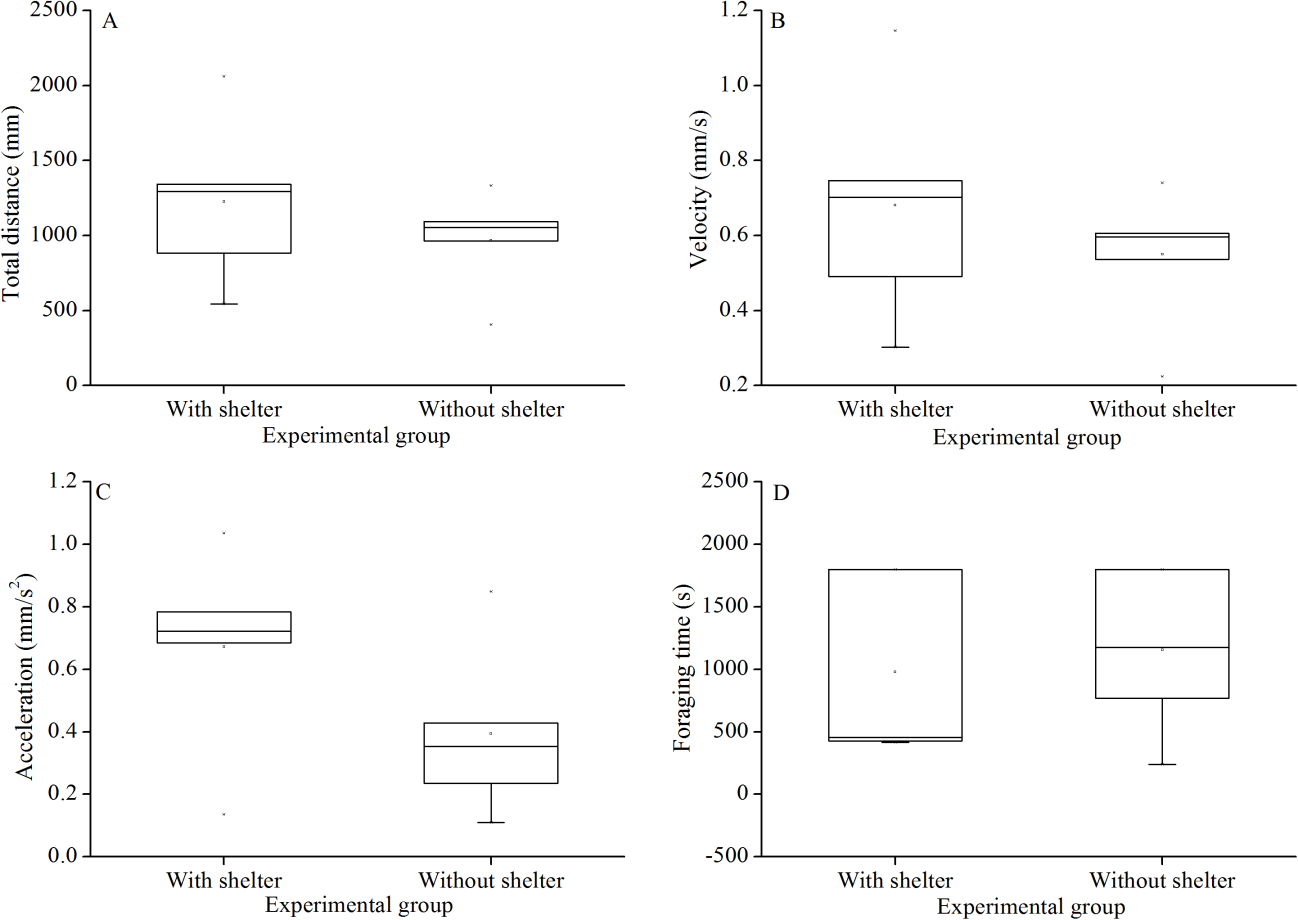
Movement and foraging behavior: total distance of movement (A), velocity (B), acceleration (C) and foraging time (D) of Glyptocidaris crenularis in different experimental groups 30 minutes after the beginning of observations (*N* = 5, mean ± SD).

Foraging behavior did not differ significantly in total distance of movement (*P* = 0.415, Fig. 4A), velocity (*P* = 0.432, Fig. 4B), acceleration (*P* = 0.191, Fig. 4C) and foraging time (*P* = 0.704, Fig. 4D) between experimental groups.

### Sheltering behavior

**Figure 5:**
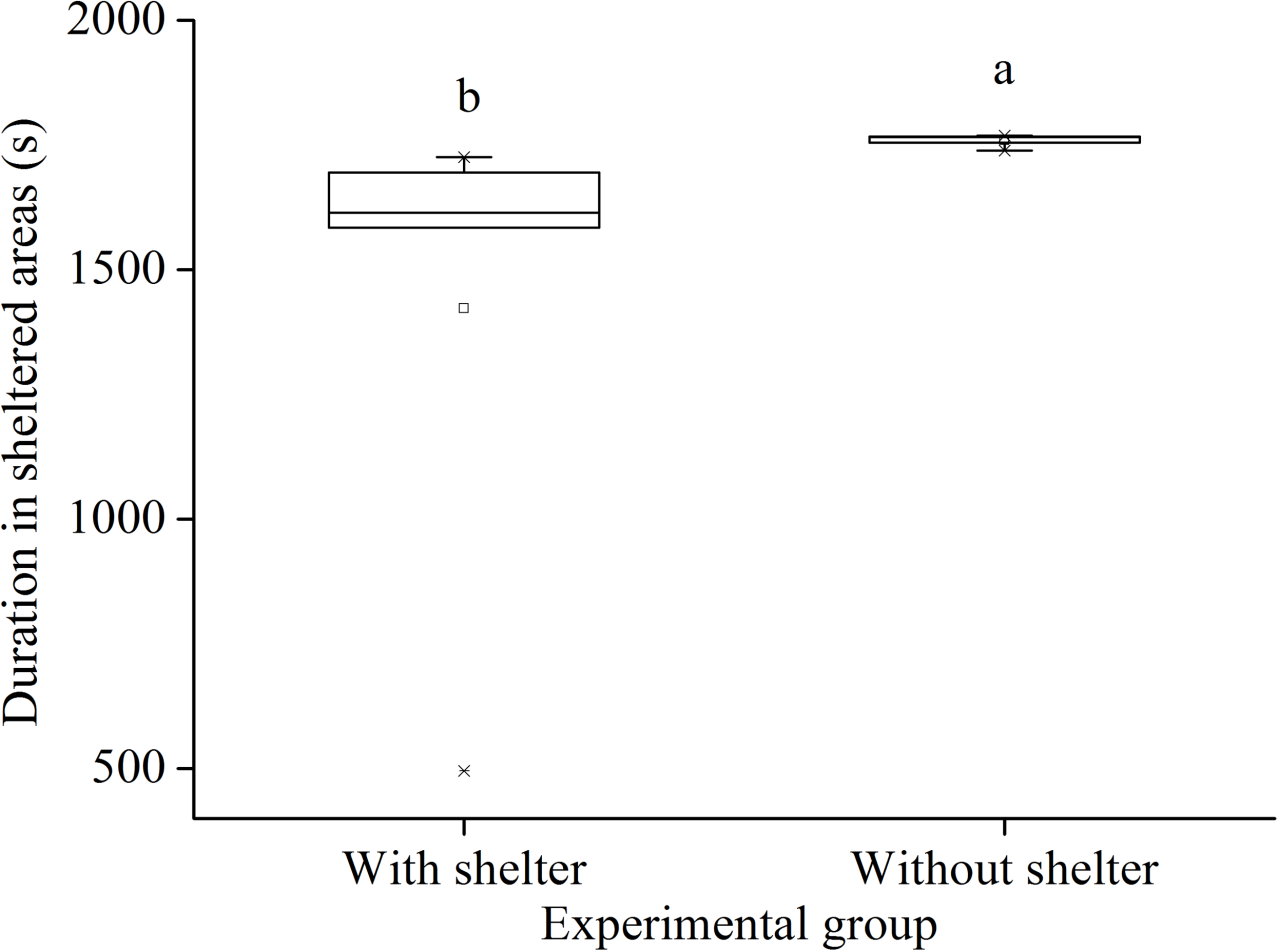
Duration in sheltered areas of *Glyptocidaris crenularis* in different experimental groups during 30 min after the beginning of experiment (*N* = 5, mean ± SD). Letters above the bars represent significance (*P* < 0.05).

In the sheltering behavior experiment after 7 year with or without shelters, sea urchins of the group with shelters spent significantly less time in sheltered areas than those of the group without shelters (*P* = 0.034, Fig. 5).

### Body size

**Figure 6:**
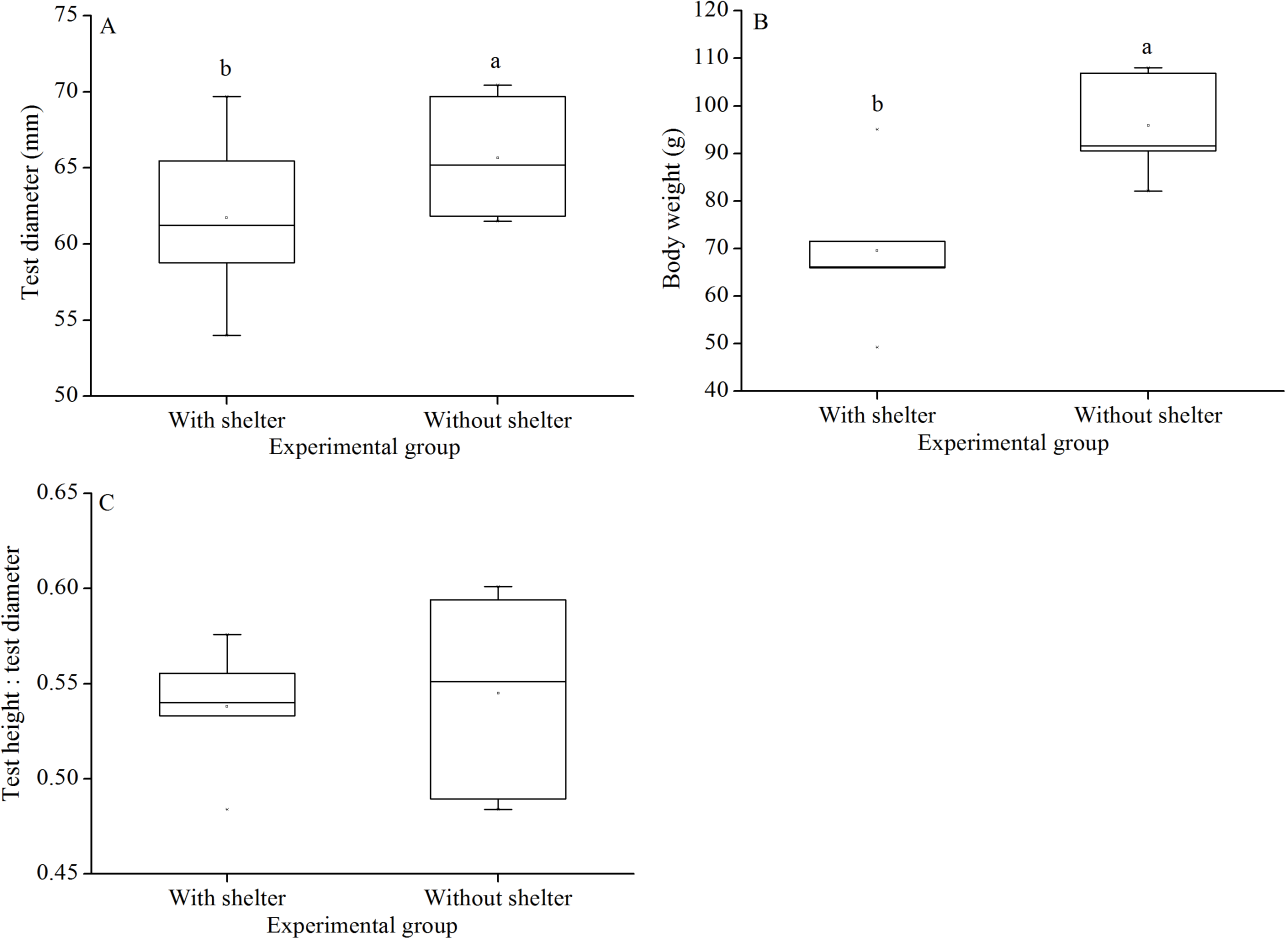
Test diameter (A), body weight (B) and test height: test diameter (C) of *Glyptocidaris crenularis* in different experimental groups (*N* = 5, mean ± SD). Letters above the bars represent significance (*P* < 0.05).

Test diameter of non-sheltered sea urchins (66.5 ± 3.2 mm) were significantly higher than that of sheltered ones (60.1 ± 3.8 mm) (*P* = 0.033, Fig. 6A). Consistently, wet body weights of non-sheltered individuals (95.8 ± 10.1 g) were significantly higher than that those of the sheltered ones (69.6 ± 14.8 g) (*P* = 0.019, Fig. 6B). There were no significant differences in test height:test diameter ratio (*P* = 0.955, Fig. 6C).

### Test thickness, test weight, lantern weight and lantern length

**Figure 7:**
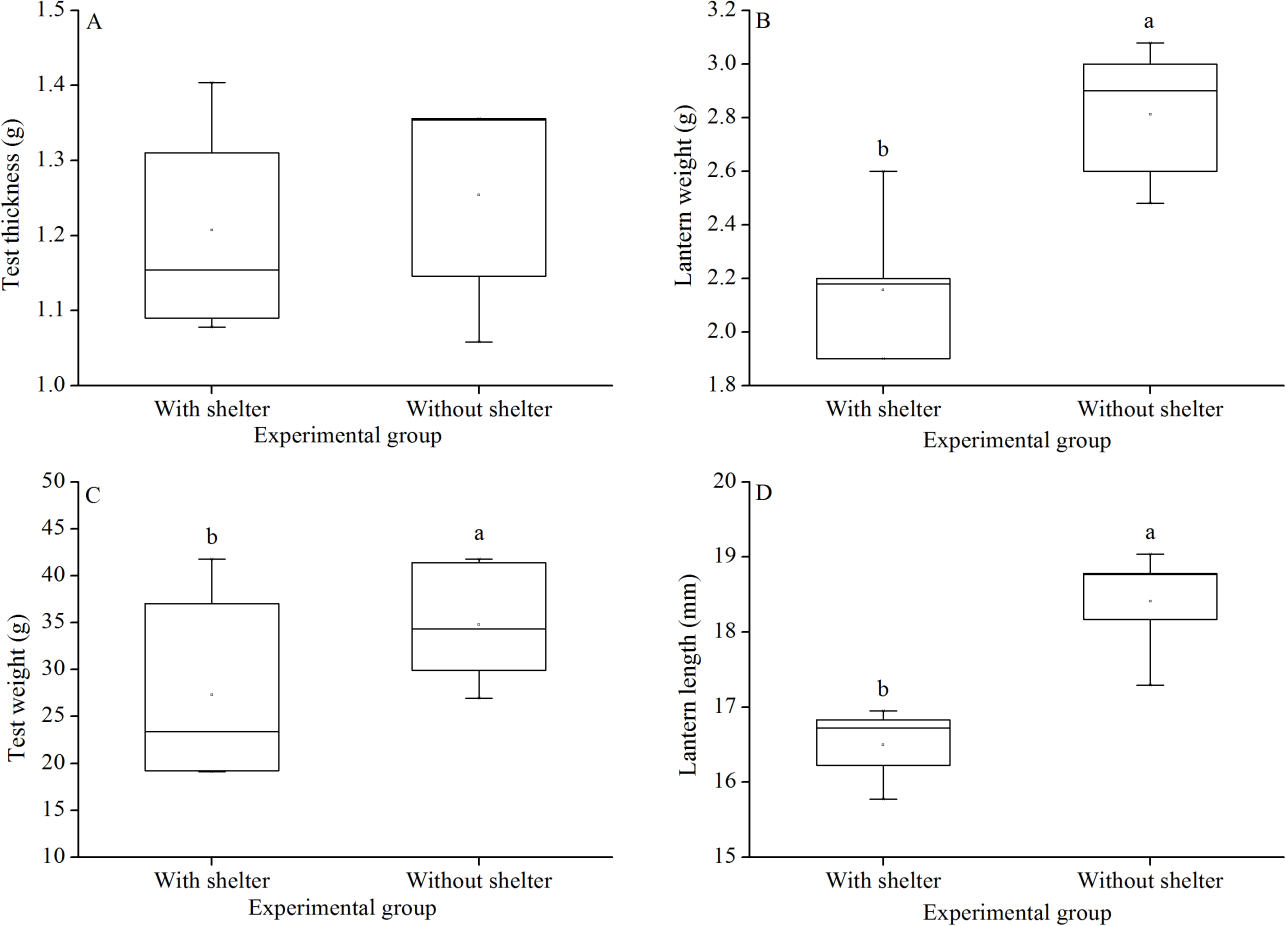
Test weight (A), test thickness (B), lantern weight (C) and lantern length (D) of *Glyptocidaris crenularis* in different experimental groups (*N* = 5, mean ± SD). Letters above the bars represent significance (*P* < 0.05).

Test thickness of *G. crenularis* showed no significant difference between the two groups (*P* = 0.619, Fig. 7A). Sea urchins of the group without shelters showed significantly greater test weight (*P* = 0.021, Fig. 7B), lantern weight (*P* = 0.005, Fig. 7C) and lantern length (*P* = 0.001, Fig. 7D) than those of the group with shelters.

### Weight of gut and gonads

**Figure 8:**
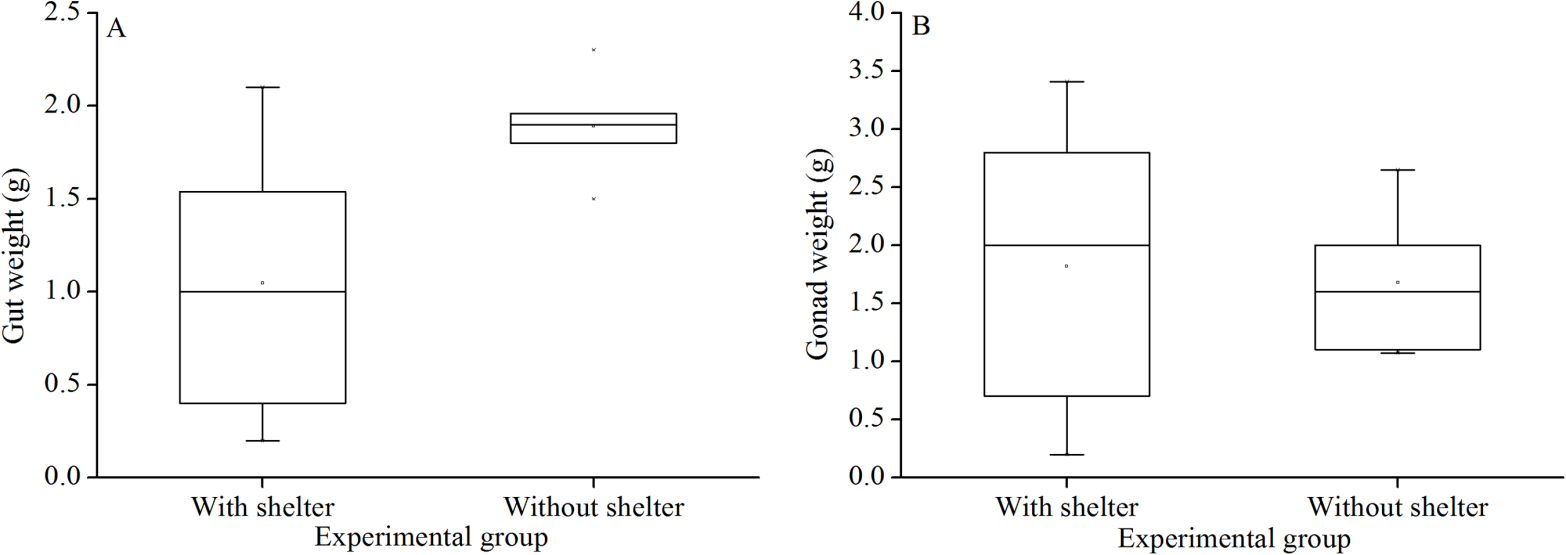
Gut weight (A) and gonads weight (B) of *Glyptocidaris crenularis* in different experimental groups (*N* = 5, mean ± SD).

There was no significant difference in gut weights (*P* = 0.055, Fig. 8A) and gonads weight (*P* = 0.843, Fig. 8B) between the groups with and without shelters.

## Discussion

Shelters are important for the fitness of many benthic marine invertebrates such as sea urchins, although the environments without shelters are ubiquitous in benthic ecosystems. Although shelters significantly improved the survival of small sea urchins *Strongylocentrotus purpuratus* (Clemente et al., 2013), the body size of sea urchins in the shelter conditions was significantly smaller (Zhao, Bao & Chang, 2016). Long-term laboratory experiments are essential to evaluate the trade-off between fitness benefits and costs (Zhao et al., 2018a; Dupont et al., 2013). Our previous study investigated fitness related traits of *G. crenularis* in shelter conditions for a duration of ~2.5 years (Zhao, Bao & Chang, 2016).

In the present study, we found significantly lower Aristotle’s lantern reflex in sea urchins in the sheltering conditions for over 7 years than those in the conditions without shelters. A potential explanation of the decreased Aristotle’s lantern reflex is the reduced neuromuscular functions for the lantern in special environments (Brothers & McClintock, 2015). Because sea urchins play an important role in intertidal and sub-tidal ecosystems (Pearse, 2006), reduced Aristotle’s lantern reflex probably decreases the overgrazing of sea urchins (Hughes, Reed & Boyle, 1987).

In addition to the Aristotle’s lantern reflex, foraging is important for feeding and food consumption of sea urchins. In the present study, there was no significant difference in foraging behavior and movement (total distance, velocity and acceleration of movement in 30 min) of *G. crenularis* with or without shelters for over 7 years. A reasonable explanation for this phenomenon is that food was available during the whole study. Significantly more sea urchins did not shelter when food was supplied outside the shelter, suggesting that sea urchins do not necessarily hide themselves when food is available (Zhao et al., 2013). The present study indicates that decreased food consumption (Zhao, Bao & Chang, 2016) is probably a result of the reduction of Aristotle’s lantern reflex, rather than reduced foraging behavior.

Surprisingly, we found that sheltering behavior of individuals with shelters was significantly lower than individual without shelters after 7 years. In the present study, the light intensity was 160.3–296.7 lx in the light areas and 13.6–47.7 lx in the sheltered areas. This result is consistent with a recent finding that the sea urchins *S. intermedius* exposed to low light intensity (~24 lx) were significantly attracted to high light intensity (~220 lx) (Sun et al., 2019). The current result indicates that long term condition suitable for sheltering behavior enhances the positive phototaxis of sea urchins.

Shelters did not significantly affect righting response time of *G. crenularis* after 7 years, compared with the condition without shelters. The current result indicates that righting behavior is relatively robust despite long-term exposure to different environmental conditions. In addition, long-term sheltering condition did not significantly affect covering behavior. This result can be explained by the finding that sea urchins preferred covering behavior over sheltering behavior (Zhao et al., 2014). In addition to sheltering behavior, righting and covering behaviors are essential to protect sea urchins from predation and hydrographic turbulence (Pinna et al., 2012; Brothers & McClintock, 2015). Thus, non-impacted righting and covering behaviors probably enhance the avoidance capabilities of sea urchins in shelter conditions to escape from adverse environments.

Body size and organs of sheltered sea urchins were significantly lower than those of non-sheltered individuals for ~2.5 years (Zhao, Bao & Chang, 2016). However, it is debatable whether the body size of sea urchins is affected in the sheltering conditions over a longer duration. Consistently, the present study found that test diameter, wet body weight, test weight, lantern length and lantern weight were significantly lower in sea urchins of the group with shelters. These results indicate that body size was negatively impacted in sea urchins of the group with shelters after 7 years. However, gut and gonad weights were not significantly affected in sheltered *G. crenularis* for over 7 years. These results indicate shelter had no effects on gonads and gut from ~2.5 years to over 7 years.

As an urchin grows, test flexibility results from the growth of producing rapid changes in height: diameter ratios and morphological plasticity in sutures between test ossicles loosen (Ellers, Johnson & Moberg, 1998; Johnson et al., 2001). There was no significant difference in test shape (test height:test diameter) between sea urchins with or without shelters for over 7 years. In contrast, cavities (as another kind of shelter) resulted in significantly reshaped test for only 8–20 weeks in the sea urchin *S. purpuratus* (Hernández & Russell, 2010). This comparison indicates test shape of sea urchins is variable in different kinds of shelters. Further, the present study showed no significant difference in test thickness between sea urchins with and without shelters after 7 years. This result is consistent with a previous finding that long-term (~10 months) elevated temperature did not significantly affect test thickness, but short-term exposure (~4 months) did (Dupont et al., 2013). The test thickness is essential to further protect sea urchin from predation and physical turbulence (Collard et al., 2016).

## Conclusion

The present long-term study suggests that the previously reported reduction in feeding rate of *G. crenularis* probably resulted from a reduction in reflexive feeding (Aristotle’s lantern reflex), rather than changes in foraging behavior. Actively seeking sheltering behavior was negatively impacted in individuals with continuous access to shelters. However, covering and righting behaviors did not differ in sheltered sea urchins, indicating that these behaviors are maintained to escape from adverse environments regardless of shelter. Body size of sea urchins was significantly lower in sheltered individuals even after 7 years. Weights of gonads and gut were not significantly different after 7 years despite previous observations of differences after ~2.5 years. However, the present study is only a laboratory investigation for one urchin species (*G. crenularis*) which does not consider the complexity of natural environments and may not occur in all sea urchin species. Field studies should be carried out in *G. crenularis* and other sea urchin species, before a more universal conclusion can be drawn.

## Supplemental Information

10.7717/peerj.8886/supp-1 Supplemental Information 1 Data of Aristotle’s lantern reflex.

10.7717/peerj.8886/supp-2 Supplemental Information 2 Data of foraging behavior.

10.7717/peerj.8886/supp-3 Supplemental Information 3 Data of sheltering behavior.

10.7717/peerj.8886/supp-4 Supplemental Information 4 Data of covering behavior.

10.7717/peerj.8886/supp-5 Supplemental Information 5 Data of righting behavior.

10.7717/peerj.8886/supp-6 Supplemental Information 6 Data of body measurements of sea urchins.

## Additional Information and Declarations

### Data Availability

The following information was supplied regarding data availability: The raw data are available as Supplemental Files.

## References

[ref-1] Abarca M, Boege K (2011). Fitness costs and benefits of shelter building and leaf trenching behavior in a pyralid caterpillar. Ecological Entomology.

[ref-2] Agatsuma Y, Lawrence JM (2013). Strongylocentrotus intermedius. Edible Sea Urchins Biology and Ecology.

[ref-3] Brothers C, McClintock J (2015). The effects of climate-induced elevated seawater temperature on the covering behavior, righting response, and Aristotle’s lantern reflex of the sea urchin Lytechinus variegates. Journal of Experimental Marine Biology and Ecology.

[ref-4] Byrne M, Smith AM, West S, Collard M, Dubois P, Graba-landry A, Dworjanyn SA (2014). Warming influences Mg^2+^ content, while warming and acidification influence calcification and test strength of a sea urchin. Environmental Science & Technology.

[ref-5] Ceccherelli G, Pais A, Pinna S, Serra S, Sechi N (2009). On the movement of *Paracentrotuslividus* towards *Posidoniaoceanica* seagrass patches. Journal of Shellfish Research.

[ref-6] Chang Y, Ding J, Song J, Yang W (2004). Biology and aquaculture of sea cucumbers and sea urchins.

[ref-7] Clemente S, Hernández JC, Montaño-Moctezuma G, Russell MP, Ebert TA (2013). Predators of juvenile sea urchins and the effect of habitat refuges. Marine Biology.

[ref-8] Collard M, Rastrick SPS, Calosi P, Demolder Y, Dille J, Findlay HS, Hall-Spencer JM, Milazzo M, Moulin L, Widdicombe S, Dehairs F, Dubois P (2016). The impact of ocean acidification and warming on the skeletal mechanical properties of the sea urchin *Paracentrotuslividus* from laboratory and field observations. ICES Journal of Marine Science.

[ref-9] Ding J, Zheng D, Sun J, Hu F, Yu Y, Zhao C, Chang Y (2020). Effects of water temperature on survival, behaviors and growth of the sea urchin *Mesocentrotusnudus*: new insights into the stock enhancement. Aquaculture.

[ref-10] Dupont S, Dorey N, Stumpp M, Melzner F, Thorndyke M (2013). Long-term and trans-life-cycle effects of exposure to ocean acidification in the green sea urchin *Strongylocentrotus droenachiensis*. Marine Biology.

[ref-11] Ellers O, Johnson A, Moberg P (1998). Structural strengthening of urchins skeletons by collagenous sutural ligaments. Biological Bulletin.

[ref-12] Foo S, Byrne M (2016). Acclimatization and adaptive capacity of marine species in a changing ocean. Advances in Marine Biology.

[ref-13] Fossat P (2014). Anxiety-like behavior in crayfish is controlled by serotonin. Science.

[ref-14] Hernández J, Russell M (2010). Substratum cavities affect growth plasticity, allometry, movement and feeding rates in the sea urchin *Strongylocentrotus purpuratus*. Journal of Experimental Biology.

[ref-15] Hughes TP, Reed DC, Boyle M-J (1987). Herbivory on coral reefs: community structure following mass mortalities of sea urchins. Journal of Experimental Marine Biology and Ecology.

[ref-16] Hyman L (1955). The invertebrates: echinodermata, the coelomate bilateria.

[ref-17] Johnson AS, Ellers O, Lemire J, Minor M, Leddy HA (2001). Sutural loosening and skeletal flexibility during growth: determination of drop-like shapes in sea urchins. Proceedings of the Royal Society of London. Series B: Biological Sciences.

[ref-18] Jones W, Powell M, Gibbs V, Hammer H, Watts S (2010). The effect of dietary selenium on weight gain and gonad production in the sea urchin, *Lytechinusvariegatus*. Journal of the World Aquaculture Society.

[ref-19] Pawson DL, Pawson DJ (2013). Bathyal sea urchins of the Bahamas, with notes on covering behavior in deep sea echinoids (Echinodermata: Echinoidea). Deep Sea Research Part II: Topical Studies in Oceanography.

[ref-20] Pearse J (2006). Ecological role of purple sea urchins. Science.

[ref-21] Pinna S, Pais A, Campus P, Sechi N, Ceccherelli G (2012). Habitat preferences of the sea urchin *Paracentrotus lividus*. Marine Ecology Progress Series.

[ref-22] Sanford E, Kelly M (2011). Local adaptation in marine invertebrates. Annual Review of Marine Science.

[ref-23] Sun J, Chi X, Yang M, Ding J, Shi D, Yu Y, Chang Y, Zhao C (2019). Light intensity regulates phototaxis, foraging and righting behaviors of the sea urchin *Strongylocentrotus intermedius*. PeerJ.

[ref-24] Tamaki H, Muraoka D, Inoue T (2018). Effect of water flow on grazing by the sea urchin (*Strongylocentrotus nudus*) in the presence of refuge habitat. Journal of Water and Environment Technology.

[ref-25] Verling E, Crook A, Barnes D (2002). Covering behavior in *Paracentrotus lividus*: is light important?. Marine Biology.

[ref-26] Zhang L, Zhang L, Shi D, Wei J, Chang Y, Zhao C (2017). Effects of long-term elevated temperature on covering, sheltering and righting behaviors of the sea urchin *Strongylocentrotus intermedius*. PeerJ.

[ref-27] Zhao C, Liu P, Zhou H, Tian X, Chang Y (2013). Diel observation on the distribution of the sea urchin *Strongylocentrotus intermedius* under different food availability and shelter conditions in the laboratory. Marine and Freshwater Behaviour and Physiology.

[ref-28] Zhao C, Tian X, Feng W, Hu L, Zhou H, Chang Y (2014). Diel observation on the trade-off between covering and sheltering behaviors of male and female *Strongylocentrotus intermedius* in laboratory. Journal of Marine Biological Association of the United Kingdom.

[ref-29] Zhao C, Bao Z, Chang Y (2016). Fitness-related consequences shed light on the mechanisms of covering behaviors in the sea urchin *Glyptocidaris crenularis*. Marine Ecology.

[ref-30] Zhao C, Zhang L, Qi S, Shi D, Yin D, Chang Y (2018a). Multi-level effects of long-term elevated temperature on fitness related traits of the sea urchin *Strongylocentrotus intermedius*. Bulletin of Marine Science.

[ref-31] Zhao C, Zhang L, Shi D, Chi X, Yin D, Sun J, Ding J, Yang M, Chang Y (2018b). Carryover effects of short-term UV-B radiation on fitness related traits of the sea urchin *Strongylocentrotus intermedius*. Ecotoxicology and Environmental Safety.

